# Muscle quantity and function measurements are acceptable to older adults during and post- hospitalisation: results of a questionnaire-based study

**DOI:** 10.1186/s12877-021-02091-3

**Published:** 2021-02-25

**Authors:** Carly Welch, Carolyn Greig, Tahir Masud, Thomas A. Jackson

**Affiliations:** 1 Institute of Inflammation and Ageing, College of Medical and Dental Sciences, University of Birmingham, Birmingham, B15 2TT UK; 2 MRC-Versus Arthritis Centre for Musculoskeletal Ageing Research, University of Birmingham and University of Nottingham, Birmingham, UK; 3 University Hospitals Birmingham NHS Foundation Trust, Birmingham, B15 2GW UK; 4 School of Sport, Exercise, and Rehabilitation Sciences, University of Birmingham, Birmingham, B15 2TT UK; 5 Birmingham Biomedical Research Centre, University of Birmingham and University Hospitals Birmingham NHS Foundation Trust, Birmingham, UK; 6 University of Nottingham, Nottingham, NG7 2RD UK; 7 Nottingham University Hospitals NHS Trust, Nottingham, NG5 1PB UK

**Keywords:** Acceptability, Sarcopenia, Handgrip, Ultrasound, Research participation, Older adults

## Abstract

**Background:**

To evaluate the acceptability of handgrip strength, gait speed, quadriceps ultrasound, and Bioelectrical Impedance Analysis (BIA) to older adults conducted during and following hospitalisation.

**Methods:**

Questionnaire-based study conducted upon completion of prospective cohort study, with follow-up in either Queen Elizabeth Hospital Birmingham (QEHB), UK, or participant’s own home following recent admission to QEHB. Outcome measures were acceptability as defined by total multi-domain score for each test (maximum score 35), and by frailty status.

**Results:**

Forty adults aged 70 years and older admitted for emergency abdominal surgery, elective colorectal surgery, or acute bacterial infections (general medicine) participated. Handgrip strength (median 33, IQR 30–35; *p* = 0.001), gait speed (median 32, IQR 30–35; *p* = 0.002), ultrasound quadriceps (median 33, IQR 31–35; p = 0.001), and BIA (median 33.5, IQR 31–35; p = 0.001) were considered highly acceptable. Participants responded positively that they enjoyed participating in these tests, and considered these tests of importance. There was no difference in scores between tests (*p* = 0.166). Individual total test scores did not differ between patients with and without frailty. Qualitative data are also presented on drivers for research participation.

**Conclusions:**

Handgrip strength, gait speed, ultrasound quadriceps, and BIA are acceptable tests to older adults during and following hospitalisation. Our results may serve as standards when evaluating acceptability of other tests.

**Trial registration:**

Prospectively registered February 2019: https://clinicaltrials.gov/ct2/show/NCT03858192

**Supplementary Information:**

The online version contains supplementary material available at 10.1186/s12877-021-02091-3.

## Background

Acceptability is a complex construct, but it is acknowledged that this can affect patient adherence both in clinical practice and research. A construct for measurement of acceptability has been proposed consisting of affective attitude, burden, ethicality, intervention coherence, opportunity costs, perceived effectiveness, and self-efficacy [1]. Sarcopenia is an area of increasing research and clinical interest. It is defined by the European Working Group on Sarcopenia 2 (EWGSOP2) as reduced skeletal muscle strength with reduced muscle quantity/quality; additional demonstration of low physical performance defines severe sarcopenia [2]. Cut-offs are taken as two standard deviations (SDs) below the mean of young healthy reference populations. Acute sarcopenia refers to acute decline in muscle quantity/quality and/or function leading to incident sarcopenia within six months, normally following a stressor event [2, 3]. EWGSOP2 recommends measurement of handgrip strength for muscle strength, and either Dual-energy X-ray Absorptiometry (DXA) or Bioelectrical Impedance Analysis (BIA) for evaluation of muscle quantity in clinical environments [2]. Ultrasonography is a recognised emerging alternative to DXA and BIA [4]. Muscle quality can also be evaluated by ultrasound echogenicity [4], or the BIA-measured phase angle [5]. EWGSOP2 recommends assessment of physical performance by Short Physical Performance Battery (SPPB), gait speed, Timed Up and Go (TUG), or 400 m walk time [2]. However, the acceptability of these measures to patients or research participants has not been previously evaluated.

### Objectives

To evaluate the acceptability of handgrip strength, gait speed, quadriceps ultrasonography, and BIA to patients, when measured as part of an observational study during and post-hospitalisation. The aim of the main study was to characterise acute sarcopenia in hospitalised older patients.

## Methods

### Participants

The main protocol for this study has been published elsewhere [6]. Our reporting is consistent with Strengthening the Reporting of Observational Studies in Epidemiology (STROBE) guidelines. Patients were recruited to one of three cohorts from the Queen Elizabeth Hospital Birmingham (QEHB) – general medical patients with infections, elective colorectal surgery, or emergency abdominal surgery. Inclusion criteria for each cohort were aged 70 years and older and hospitalised (or expected to be hospitalised for the elective cohort) for an acute bacterial infection, major colorectal surgery procedure, or emergency abdominal surgery procedure. Exclusion criteria were the inability to understand verbal and written English, or imminently dying. Informed consent or personal consultee declaration was obtained for all participants. Medical patients were recruited within 48 h of admission, emergency surgery patients were recruited pre-operatively or within 48 h post-operatively, and elective surgery patients were recruited in pre-operative assessment clinic.

### Study design

Quadriceps ultrasound, BIA, handgrip strength, and physical performance (either SPPB or gait speed alone depending on cohort and timing of assessment) were measured serially as part of this study. These were performed within 48 h of admission/surgery, within one week of admission/surgery, and three months after admission/surgery. In the elective cohort, measurements were also performed prior to admission.

### Outcome measures

Quadriceps ultrasound was performed anteriorly over both thighs at the midpoint between the greater trochanter, and the joint line of the knee. Participants were positioned with their knees in natural relaxation, with a firm wedge below the knees, and the upper body reclined to 45^o^. Contact gel was applied to the skin and measurements were taken using a linear probe using a Venue 50 device (GE Healthcare). A minimum of three measurements were taken on each side; a fourth was taken if rectus femoris (RF), vastus intermedius (VI), or subcutaneous (SC) measures varied by more than 10% between each other. These measures were used to calculate the Bilateral Anterior Thigh Thickness (BATT – right RF + left RF + right VI + left VI) [4]. BIA was performed in the same position by applying electrodes to the right hand and foot and recording measures using a Bodystat Quadscan 4000 as per the manufacturer’s instructions. BIA was not performed in participants with implanted cardiac devices. Handgrip strength was measured using a Jamar dynamometer; participants sat in a chair with their elbow flexed at 90^o^ and advised to squeeze as hard as they could [7]. Two readings were taken on each side. Gait speed was measured over a 4 m course; participants were advised to walk at a normal comfortable pace, using walking aids if necessary.

### Frailty

Frailty was defined dichotomously (frail vs. non-frail) according to the phenotype definition [8] at the point of the completion of acceptability questionnaire. Frailty was defined as scoring three or greater of weight loss (recorded or self-report), low handgrip strength, low walking speed, self-reported exhaustion, or low physical activity, as detailed in the main study protocol [6].

### Acceptability evaluation

An acceptability questionnaire was developed, as described in the main study protocol [6] and online supplement, which asked participants to state how highly they agreed with positive statements about seven different aspects of acceptability for each of handgrip strength, 4 m gait speed, ultrasound quadriceps, and BIA (Table 1) [1]. The questionnaire was completed by the same researcher who administered the muscle quantity and function assessments. We evaluated gait speed alone rather than SPPB to ensure consistency across cohorts, and prevent burden to participants from the acceptability evaluation. Responses were given using a Likert scale (1 = strongly disagree, 2 = disagree, 3 = neither agree nor disagree, 4 = agree, 5 = strongly agree). Participants were also able to provide additional comments related to the study in general or any study-related procedures. This questionnaire was administered to all participants at the point of their three-month follow-up, in either their own home, or the Inflammation Research Facility, QEHB. Recruitment was paused due to the Coronavirus 2019 (COVID-19) pandemic and the protocol was later amended to remove in-person follow-up at three months, to reduce unnecessary contact with vulnerable participants. This sub-study includes participants who were recruited prior to this amendment.

**Table 1:** Positive statements included in acceptability questionnaire and applicable domains. Participants were asked to rate their agreement with these statements on a scale from 1 = strongly disagree to 5 = strongly agree

Acceptability domain	Statement
Affective attitude	I enjoyed participating in this test
Burden	Minimal effort was required to complete this test
Ethicality	This test was unobtrusive
Coherence	I understand how this test works and its importance
Opportunity costs	This test was not time-consuming
Perceived effectiveness	This test is likely to have a positive impact on patients
Self-efficacy	I felt confident that I could complete this test

### Statistical analysis

Data were imported into IBM SPSS Version 26. Counts for each Likert score were derived and presented visually with horizontal bar charts. For each outcome measure, a total score was derived for all acceptability domains for each participant (minimum possible score 7, maximum possible score 35). Median total acceptability scores were calculated for each outcome. We used one-sample Kolmogorov-Smirnov normal tests to evaluate distributions of total acceptability scores for each outcome. We used the Friedman test to assess differences in total acceptability scores between outcome measures and Mann-Whitney U tests to assess for differences in individual total scores between those with and without frailty. The main study was powered for a different primary outcome. A post-hoc power calculation showed that a sample size of 40 was able to detect a difference in total acceptability score of 1.35, with 80% power and 5% alpha, assuming a null hypothesis median score of 20 (i.e. neither agree nor disagree selected for all answers) and an expected normal distribution.

### Qualitative analysis

Free text comments were transcribed by the researcher (CW) linked against their identifiable study number. The researcher (CW) familiarised themselves with the comments and identified emergent themes. Thematic analysis was conducted using an inductive approach, with no prespecified hypotheses of what data may arise from these comments. The participant details were linked to text after identification of emergent themes. Consensus agreements of themes was reached by researchers not involved in initial transcription and data reduction.

### Public involvement

Patients and members of the public were extensively involved in the planning and development of the main study. The questionnaire used in this study was developed with direct involvement of healthy older adults. The results of this study itself will be of direct relevance to future studies and clinical practice involving the measures described.

## Results

**Fig. 1:**
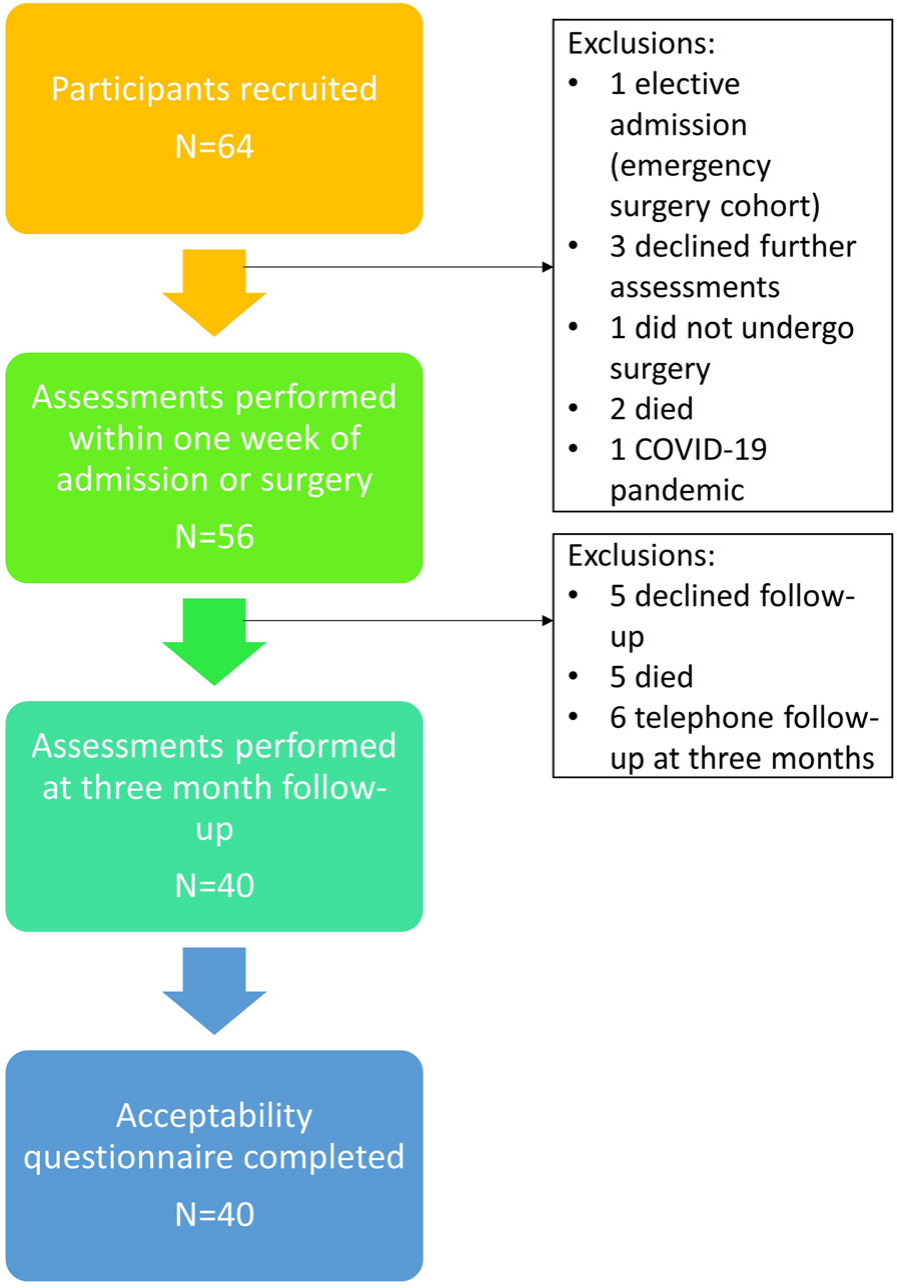
Recruitment and follow-up of participants within main study

Sixty-four participants (24 elective surgery, 24 general medical, 16 emergency surgery) were recruited to the main study from May 2019 to March 2020. Figure 1 shows the recruitment and follow-up flowchart of included participants. Forty participants (17 elective surgery, 13 general medical, 10 emergency surgery) were followed-up in person at three months and all completed the acceptability questionnaire. The characteristics of participants who completed the questionnaire are shown in Table 2.

**Table 2:** Characteristics of participants who completed acceptability questionnaire

		Study population (*N* = 40)
Age – mean (SD)		78.1 (6.3)
Sex – % females (N)		47.5 (19)
Ethnicity	White British or White Irish – % (N)	95.0 (38)
	Indian – % (N)	5.0 (2)
Phenotypic frailty at follow-up – % frail (N)		57.5 (23)
Gait speed – mean (SD)		0.67 (0.28)
Handgrip strength – mean (SD)	Males	25.8 (10.8)
	Females	16.9 (8.0)

### Quantitative results

**Fig. 2:**
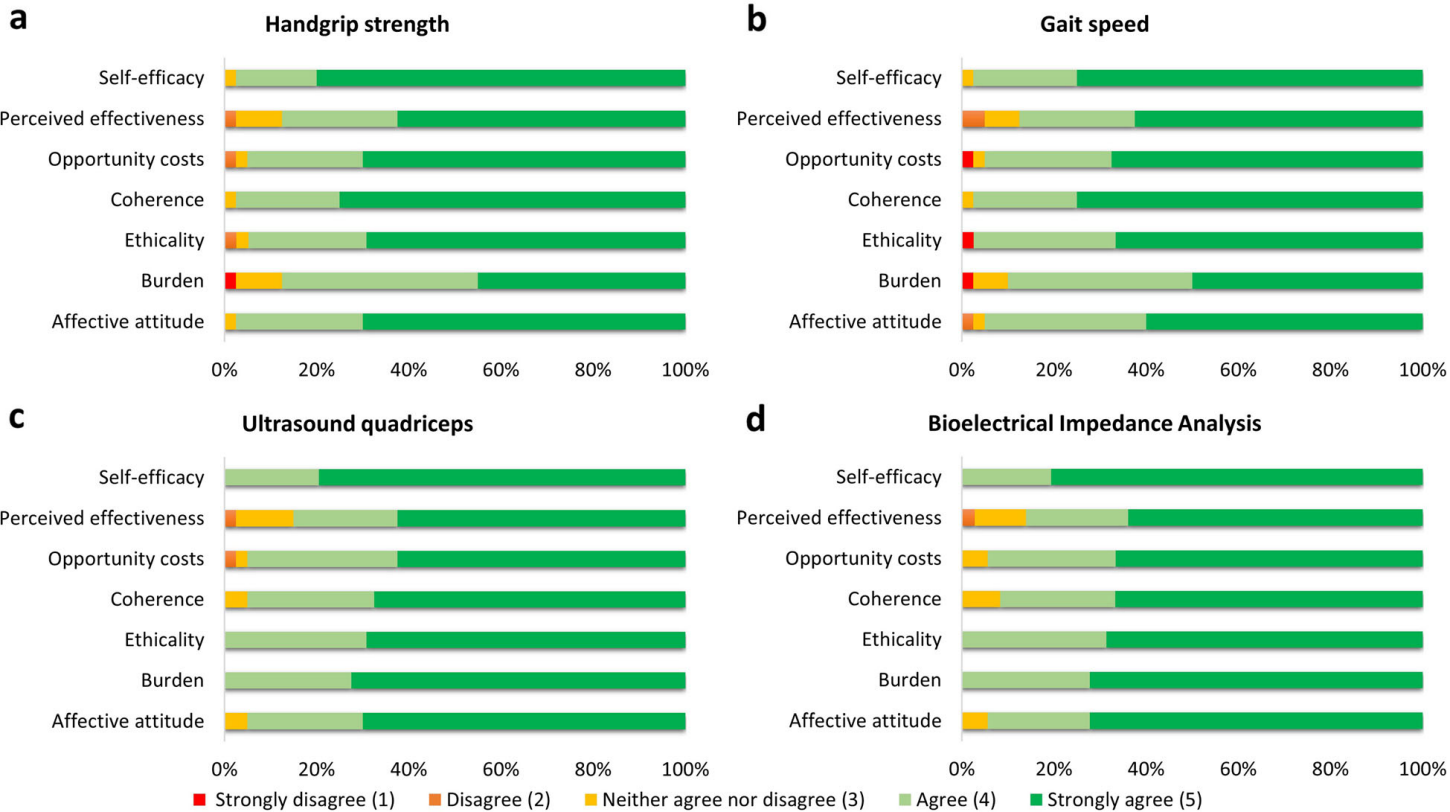
Distributions of individual responses for each acceptability domain for each outcome measure

Figure 2a-d shows the distribution of response scores for each acceptability domain for each outcome measure. Overall, domains rated highly for all outcome measures, with the majority of participants stating that they agreed or strongly agreed with each positive statement for each outcome. The domain with the least agreeability was burden for both handgrip strength and gait speed; some participants disagreed with the statement that minimal effort was required to complete these tests. The domain with the highest agreeability was self-efficacy, particularly for ultrasound and BIA; participants agreed or strongly agreed that they enjoyed participating in these tests.

Table 3 shows the median overall scores for each outcome measure, separated by phenotypic frailty. All total score distributions were individually significant. However, total scores did not significantly differ between outcome measures. Additionally, scores did not significantly differ for outcomes between those with and without frailty.

**Table 3:** Median test scores for total acceptability scores for each outcome measures overall and divided by phenotypic frailty status. The minimum possible total median score was 7, and the maximum possible score was 35. Higher scores suggest higher levels of acceptability

		Median (IQR)	*p*-value (one sample)	p-value (groups)
**Overall**				
Handgrip strength (N = 40)		33 (30–35)	0.001	0.166
Gait speed (N = 40)		32 (30–35)	0.002	
Ultrasound quadriceps (N = 40)		33 (31–35)	0.001	
Bioelectrical Impedance Analysis (*N* = 36)		33.5 (31–35)	0.001	
**Frailty**				
Handgrip strength	Frail (*N* = 23)	33 (30–33)	0.052	0.396
	Non-frail (*N* = 17)	34 (30–35)	0.030	
Gait speed	Frail (N = 23)	32 (28–32)	0.031	0.242
	Non-frail (N = 17)	34 (31–35)	0.019	
Ultrasound quadriceps	Frail (N = 23)	32 (29–32)	0.042	0.386
	Non-frail (N = 17)	34 (31–35)	0.008	
Bioelectrical Impedance Analysis	Frail (*N* = 20)	32 (29–32)	0.009	0.352
	Non-frail (*N* = 16)	34 (32–35)	0.008	

### Qualitative results

#### Study procedures

Many participants commented positively on their experience of completing the study-related procedures. Some participants commented that they enjoyed completing the tests, in that they gave them something new to try, and additional knowledge about their health.

*“I was looking forward to it actually [gait speed]; I thought at least it would get me moving …”*

*“Actually, I enjoyed doing all the tests”*

*“It was welcoming really to try to do things that I couldn't do 12 months ago”*

*“Anything positive to do with your health is definitely a good thing”*

*“It was quite relaxing”*

*“I'm pleased with how I've done. I enjoyed it all”*

Participants also expressed agreement with the ethicality, coherence, and perceived effectiveness of the study procedures.

*“All of the testing has been unobtrusive and seemed very sensible”*

*“It's very important … it's important for people in the future …. All tests like you do are important … Future generations have still got to get old”*

*“They're all very worthwhile and very good”*

*“Perfectly alright … It's all good to have these tests as you don't know yourself”*

### Other procedures

Some participants expressed that other aspects of the SPPB, the acceptability of which were not formally assessed in this study, were more burdensome.

*“Apart from ‘getting up from the chair’ [chair stands] it was no effort”*

*“The only one that really got me was ‘the chair’ [chair stands]”*

*“The only thing was the balance thing [tandem stand]”*

### Research participation

Although not the primary focus of this study, participants expressed comments relating to their reasons for participating in research. Common themes that emerged were around the desire to help others and feeling that they had been able to provide a service.

*“If it helps anyone else to get better then so be it”*

*“I do them because I know that I'm helping to improve things”*

*“If it's gonna be useful to you and to someone else that's good enough for me”*

*“I'm glad to be of service to someone – whatever helps you and your research”*

*“I'm glad that I was able to help …”*

Other participants expressed that they felt they had been able to learn things through participating in research, which had benefitted them personally.

*“I just find I learn something and you learn something. My motivation is I want to see the boundaries pushed back”*

*“We found out how these things work”*

*“I enjoyed it all – interesting and educational”*

*“I've just been really interested in what you've done”*

Providing the option of being able to have follow-up conducted in the participants’ own homes was also considered very positively.

*“Grateful to visitors - we enjoyed”*

*“I've enjoyed you coming and seeing you … it gives you an insight into what's going on”*

*“I'm pleased that you're able to come to me and I've not got to travel anywhere …”*

## Discussion

This is the first study to formally evaluate the acceptability of handgrip strength, gait speed, quadriceps ultrasonography, and BIA in older adults with or without frailty during and following hospitalisation. Overall, our results showed that all tests were very acceptable to participants. Our muscle quantity/quality assessments (ultrasonography and BIA) were at least as acceptable as muscle function assessments (handgrip strength and gait speed). If anything, there was a suggestion of increased perceived burden with muscle function assessments, which relates to these tests requiring the participant to actively initiate the test. Importantly, no difference in acceptability was demonstrated with frailty. This is important, as these tests are often used to evaluate frailty, and it is important that there is not a bias against participation of frail older adults in testing. However, as acceptability scores were very positive overall for all groups and all tests, the margin of any difference would be very small.

Coherence was scored high across all outcomes; this was concordant with qualitative responses, with a recurrent emerging theme that participants considered these measures to be important. Interestingly, there were no obvious variations in opportunity costs between tests, which relates to the participants’ perceptions of how time-consuming the tests were. Handgrip strength, gait speed, and BIA are certainly quicker to administer than ultrasound. However, there was no suggestion that participants considered any tests any more time-consuming than others; participants considered the time taken to complete each assessment acceptable. Acceptability of aspects of the SPPB other than gait speed were not formally examined as part of this study. However, there was a suggestion from our qualitative results that the other parts of the SPPB (balance and chair stands) may be considered more burdensome to participants. This is important as this may affect compliance with these aspects of the tests i.e. if participants recall that these parts of the test were burdensome on previous testing they may be less likely to agree to repeat them on subsequent testing. Nonetheless, we consider these results vitally important in demonstrating that all tests were at least as acceptable as each other. We consider these to be valuable results towards integration of these measures into clinical practice, and in development of future clinical trials and studies. The results of our studies may also serve as standards when assessing acceptability of other tests in similar populations e.g. muscle biopsies.

Although the purpose of this study was to determine acceptability, our qualitative results considering research participation are of relevance towards planning future clinical trials/studies in older people. Drivers for participation in research were altruism (wanting to help patients in the future), feeling that they were “giving back” towards the hospital (being of service), and the opportunity to learn/develop their own knowledge. These reasons are consistent with motivators that have been demonstrated elsewhere [9, 10]. The option for the study to be performed in participants own homes was reviewed positively. Where practical, this should be considered within study protocols involving older adults. Further research evaluating reasons why patients don’t take part in research would be of further value in ensuring that research participation is representative of the patient population.

### Study strengths

This is the first study to specifically evaluate the acceptability of the measures described to patients during and post- hospitalisation. The questionnaire devised for this study was multi-faceted and developed from recognised domains within acceptability [1]. The simplicity of the survey ensured high completion rates, enabling gathering of both quantitative and qualitative results. Additionally, obtaining feedback at the end of study completion enabled participants to have appropriate time to really consider their feedback on participation, and to be able to provide this in a comfortable environment (either their own home or a quiet clinic room). At this stage participants had also completed the assessments multiple times so were familiar with the tests. This ensured higher completion/response rates.

### Study limitations

We acknowledge that there are a number of limitations to this study. There is no agreed standard way of assessing acceptability of a medical test. Firstly, the questionnaire itself was devised by the study team. It is unknown how these results would compare against other tests that are commonly used in clinical practice i.e. we do not know whether these results represent “above average” acceptability. Additionally, perceived acceptability of tests may be biased by the agreement of participants to participate in the study in the first place and to complete follow-up; patients who refused to participate and those who did not complete follow-up may have responded differently. Unfortunately, this is an inevitable bias of any study that aims to assess acceptability via participant responses; it would not be possible to assess acceptability of a study procedure in a participant who had not agreed to participate. Results may also be biased by the fact that the questionnaires were administered by the same researcher who conducted the muscle quantity and function assessments; participants may have wished to ingratiate themselves with the research team [11]. We also acknowledge that religious or cultural differences may affect the results of this study. The majority of participants were White British or Irish and we did not collect personal information about religious beliefs. Acceptability of tests may be viewed differently in other groups e.g. individuals of some religious backgrounds may consider quadriceps ultrasonography to be more personally obtrusive [12]. As described, feedback was obtained after the participants’ final follow-up assessments, although we consider this a strength, this can also be considered a limitation. Participants may have responded differently if they had been asked to complete feedback in hospital. It is important to consider that some participants had cognitive impairment and were unable to recall the initial tests, which did not obviously affect responses.

## Conclusions

The results of this study may serve as standards for future acceptability studies e.g. when evaluating the acceptability of muscle biopsies. Handgrip strength, gait speed, BIA, and US quadriceps are acceptable to tests to older adults when performed during and after hospitalisation. This applies to those with and without frailty. We recommend the integration of these tests into clinical practice and future research, where these are considered of clinical utility.

## Supplementary Information

**Additional file 1.**

## Data Availability

The anonymised dataset is available from the corresponding author upon reasonable request.

## Abbreviations

BATT: Bilateral Anterior Thigh Thickness
BIA: Bioelectrical Impedance Analysis
COVID-19: Coronavirus 2019
DXA: Dual energy X-ray Absorptiometry
EWGSOP2: European Working Group on Sarcopenia in Older People 2
QEHB: Queen Elizabeth Hospital Birmingham
RF: Rectus Femoris
SC: Subcutaneous
SD: Standard Deviation
SPPB: Short Physical Performance Battery
STROBE: Strengthening the Reporting of Observational Studies in Epidemiology
TUG: Timed Up and Go
VI: Vastus Intermedius
